# Engagement in dance is associated with emotional competence in interplay with others

**DOI:** 10.3389/fpsyg.2015.01096

**Published:** 2015-07-31

**Authors:** Eva Bojner Horwitz, Anna-Karin Lennartsson, Töres P. G. Theorell, Fredrik Ullén

**Affiliations:** ^1^Department of Public Health and Caring Sciences, Uppsala UniversityUppsala, Sweden; ^2^Center for Social Sustainability, Department of Neurobiology, Care Sciences and Society, Karolinska InstitutetStockholm, Sweden; ^3^Department of Neuroscience, Karolinska InstitutetStockholm, Sweden; ^4^Stress Research Institute, Stockholm UniversityStockholm, Sweden

**Keywords:** alexithymia, creative achievement, dance, embodiment, emotional competence

## Abstract

This study has explored the relation between dance achievement and alexithymia in a larger Swedish population sample (Swedish Twin Registry) with a study sample of 5431 individuals. Dance achievement (CAQ) was assessed in relation to Alexithymia (Toronto Alexithymia Scale, TAS-20) including the three subscales: Difficulty Identifying Feelings (DIF), Difficulty Describing Feelings (DDF), and Externally Oriented Thinking (EOT). The results show a significant negative association between the TAS subscale (EOT) and creative achievement in dance. A high EOT score corresponds to poor ability to communicate feelings to the environment. There was no consistent association between the other factors DIF and DDF and dance achievement. Dance activity and training seem to be involved in the body’s emotional interplay with others. Embodied cognition, emotional perception, and action are discussed as factors relevant to measuring the skill of a dancer.

## Introduction

Dance is a fundamental form of human emotional expression (Krantz et al., 2006). During the past decade it has been in focus in cognitive and neural sciences as well as in social sciences in the development of our understanding of social cognition and human behavior (Brown and Parsons, 2008; Reynolds et al., 2011; Sevdalis and Keller, 2011; Bläsing et al., 2012). In relation to non-verbal communication and social behavior, it is important to be able to process biological motion by living organisms visually (Pavlova, 2012). Studies using brain-imaging techniques indicate that both cognitive and sensorimotor regions are involved in the processing of dance (Calvo-Merino et al., 2005; Cross et al., 2006; Brown and Parsons, 2008; Koch and Fuchs, 2011). Observing the actions of others can induce activity in both motor and emotional systems of the brain (Rizzolatti et al., 2001; Rizzolatti and Craighero, 2004; McGarry and Russo, 2011) and it has been suggested that this may also affect different types of empathic behavior (McGarry and Russo, 2011). The multiple aspects of embodied cognition through dance (involving both performing and perceiving processes) may help us understand in what way creative aspects of dance achievement can affect emotional competence.

One way to evaluate emotional competence is to use the construct of “alexithymia” which was introduced by Sifneos (1973) who observed that patients with psychosomatic disorders had difficulties in recognizing and finding appropriate words for emotions and feelings. In Greek the word alexithymia means “no words for feelings.” In a working age Finnish non-clinical population, the prevalence of alexithymia has been shown to be 5–10% for women and 9–17% for men (Mattila et al., 2007). It is normally distributed in the general population (Salminen et al., 1999). The prevalence of alexithymia is more frequent in chronic pain patients, 47% (Mendelson, 1982). Other health outcomes related to alexithymia are depression (Honkalampi et al., 2000) and hypertension (Jorgensen and Houston, 1986 and Grabe et al., 2010). Early stage hypertensive subjects have also shown poor ability to describe feelings (Theorell et al., 1984). Although alexithymia is not a diagnostic disorder presented in ICD-10 or DSM VI, it is suggested as a clinically relevant construct.

Alexithymia is related to impairment of emotional awareness/emotional regulation and according to the theory, it is a disability to represent emotions mentally (Taylor et al., 1997). The Toronto Alexithymic Scale (TAS; Bagby et al., 1994) is the most commonly used instrument to assess alexithymia and consists of 20 questions (Bagby et al., 2014). TAS-20 uses three different subscales: (1) difficulty identifying feelings, (2) difficulty describing feelings, (3) difficulties with externally oriented thinking. All three subscales focus on deficits in the cognitive processing of emotions, which are assumed to result in poorly regulated emotions (Taylor et al., 1991, 2014).

Thompson (1994) describes emotional regulation as the external and internal process through which an individual becomes aware of emotional reactions to be able to reach goals. Piaget’s model of different stages of cognitive development from the sensorimotor to the operational level via reflexion, describes individual differences in the ability to regulate affects (Lane and Schwartz, 1987). An individual who can operationalize and symbolize emotional responses can regulate emotions and is “less alexithymic.” The three subscales of TAS-20 may therefore be useful to measure in relation to Piaget’s model and to evaluate their relationship to creative achievement in dancing.

Embodied cognition, perception, and action are factors relevant to measuring the skill of a dancer. Dance achievement involves motor, emotional, visual, sensory, and intellectual processes. Dance may therefore facilitate differentiation of emotions (Bojner Horwitz, 2004). In a previous study, we have followed changes in movement patterns after dance in relation to creativity and quality of life, using video interpretation technique (Bojner Horwitz et al., 2003). The creative and more fluently nuanced changes in movement patterns after the dance sessions were related to an increase in patients’ differentiation of feelings. The link between dance and emotions has been in focus in several additional studies (Koch and Fuchs, 2011; McGarry and Russo, 2011) but no study has explored the relation between dance achievement and alexithymia in a larger population sample, which is the purpose of our study.

Our hypothesis is that there will be a negative association between alexithymia and creative achievement through dance in a sample representing the Swedish population. Note that this does not necessarily imply a causal relation in either direction.

## Materials and Methods

### Participants

Data were collected as part of a web-survey sent out to a cohort of approximately 32,000 twins born between 1959 and 1985 – the STAGE cohort (Lichtenstein et al., 2002) – from the Swedish Twin Registry (STR), one of the largest registries of its kind (Formann and Piswanger, 1979; Lichtenstein et al., 2002; Magnusson et al., 2013). The present study received approval from the Regional Ethics Review Board in Stockholm (Dnr 2011/570-31/5, 2011/1425-31, 2012/1107/32). In total, 11,543 individuals participated in the web-survey. Of these, 6827 individuals (1396 complete twin pairs and 4035 single twins without the co-twin) had answered the Dance achievement subscale of CAQ (see section Measures). To control for relatedness within the sample, one twin from each of the 1396 pairs was randomly selected. These 1396 individuals and all the 4035 single twins were included in the present study. Thus the study sample consisted of 5431 individuals out of 2279 were men and 3152 were women. The participants were aged between 27 and 54 (mean 41 years, SD: 8).

### Measures

#### Dance Achievement

Dance achievement was assessed using the Dance subscale of the Swedish adaptation of the Creative Achievement Questionnaire of Carson (Carson et al., 2005; Power et al., 2015). Dance creative achievement is self-rated using a 7-graded scale with the question: “How engaged are you in dance?”: 1 = “I am not dancing at all,” 2 = “I am self-taught and dancing, but I have never danced in front of an audience,” 3 = “I had dance lessons, but I have never danced in front of an audience,” 4 = “I have participated in public dance show in my place of origin, but I have never been paid for this,” 5 = “I have participated in public dance show in my place of origin, and I have been paid for this,” 6 = “I am a professional dancer,” and 7 = “I am a professional dancer, and have been reviewed by nation vide media in Swedish or international specialist press, and/or have been awarded with at least one prize for my dance.” The subjects were divided into three groups depending on their answer: Non-dancers (response 1), Amateur dancers (responses 2–4) and Professional dancers (responses 5–7).

#### Alexithymia

A back-translated and psychometrically tested Swedish version of Twenty-Item TRS (TAS-20; Bagby et al., 1994, 2014; Simonsson-Sarnecki et al., 2000) was used to measure alexithymia. TAS-20 has three subscales: Difficulty Identifying Feelings subscale (DIF) measuring difficulties to identifying emotions (five items); Difficulty Describing Feeling subscale (DDF) measuring difficulties to identify emotions (seven items), and Externally Oriented Thinking subscale (EOT) measuring a tendency to focus attention externally (eight items). The items are rated on a scale from 1 to 5 where 1 is equivalent to strongly disagree and 5 is equivalent to strongly agree. Five items are negatively keyed and back-transformed when calculating sums. The total alexithymia score is the sum of responses to all 20 items and possible scores range from 20 to 100 with higher scores indicating higher degrees of alexithymia. Cut-offs in TAS-20: scores equal to or less than 51 = non-alexithymia, 52–60 = moderate alexithymia, equal to or greater than 61 = clinical alexithymia. TAS-20 can also be used as a continuous variable when studying degrees and tendencies of alexithymia in different groups. The subscales measuring different aspects of alexithymia can range from 5 to 25 for DID, 7 to 35 for DDF, and 8 to 40 for EOT subscale. Higher scores indicating higher degrees of corresponding aspect of alexithymia. Subscales are used as continuous variables.

#### Education

Level of education was assessed by a 10-graded scale reflecting the level of formal school education according to Statistics Sweden. The lowest level was unfinished elementary compulsory school and the highest level an academic doctoral degree. The four lower levels corresponded to no more than high school education (Low education) whereas the six upper levels corresponded to at least some exposure to college or university education (High education).

#### Exposure to Culture during Childhood/Adolescence

Participants also reported how often they attended culture activities such as for example concerts and theater shows during their childhood and adolescence, choosing between “Once a year or more seldom,” “Two to eleven times per year,” “One to three times per month” and “One to several times per week.” Eighteen percentage of the participants reported that they attended culture activities two or more times per year.

#### Statistical Analyses

Percentage of males and females who reported that they danced were calculated. Percentage of the male and female dancers who were amateur dancers and professional dancers, respectively, were also calculated. Age was compared between the non-dancers and amateurs and professionals, respectively, using *t*-test. Chi-square test was used to compare the proportions of participants who (1) were men, (2) had high education and (3) attended cultural activities more than once a year during their childhood/adolescence between the non-dancers and the amateur dancers and professional dancers, respectively. Using a *t*-test, alexithymia scores were compared between men and women and between those with higher and those with lower education. The Pearson correlation was computed between age and alexithymia scores in men and women separately. Percentage of cases of moderate alexithymia and clinical alexithymia were calculated in the non-dancers, amateur dancers, and the professional dancers, separately in men and women. Four ANCOVAS were performed separately in men and women with different dependent variables: (1) the alexithymia full-scale (TAS-20) and the three subscales assessing, (2) difficulty recognizing, (3) difficulty describing and, (4) externally oriented thinking. Age and education level were entered as covariates in each analysis and Dance achievement groups (Non-dancers, Amateur dancers, and Professional dancers) were entered as independent variable. The Non-dancers group was entered as reference group. The male and female halves of the study population were regarded as independent populations. Since there were eight comparisons for men and eight comparisons for women, our significance levels were adjusted (according to Bonferroni) using the *p* = 0.05/8 = 0.006 as significance limit.

## Results

### Dance Achievement among the Participants

Of the 5431 participants in the study, 26.5% of the males and 46.1% of the females reported that they danced. Of those who reported that they danced, 97% were amateur dancers and 3% were professional dancers (in both the male and female sample). **Table 1** displays the number of participants divided into groups according to dance achievement. About 70% of the dancers were women. Having attended cultural activities more than once a year during childhood/adolescence and higher education level was more common among the dancers (amateurs and professionals) than non-dancers.

**Table 1:** Non-dancers, amateur dancers, and professional dancers in the sample of total 5431 individuals.

	Non-dancers *N* = 3374	Amateur dancers *N* = 1987	Professional dancers *N* = 70	*P*-value^a^	*P*-value^b^
Proportion of total	62.1%	36.6%	1.3%	na	na
Mean age (SD)	41 (8)	41 (8)	40 (8)	0.468	0.178
Men	49.7 %	29.3%	28.6%	<0.001	0.001
High education	60.7%	67.8%	71.0%	<0.001	0.104
Attended cultural activities more than once a year during childhood/adolescence	15.3%	23.0%	39.1%	<0.001	<0.001

*Mean (SD) or percent when indicated. *t*-test and Chi-Square test were used to compare the groups.*

*^a^Comparison between Non-dancers and Amateur dancers.*

*^b^Comparison between Non-dancers and Professional dancers.*

### Alexithymia among the Participants

As expected, women had lower alexithymia scores than men [41.8 and 46.4, respectively; *t*(5429) = 16.8, *p* < 0.001]. Among the males, 22.6% scored moderate alexithymia and 7.8% scored clinical alexithymia. Among the females, the corresponding percentages were 13.2 and 4.4%. Individuals with higher education had lower alexithymia scores than individuals with lower education [45.3 and 48.0, respectively, in men, *t*(2263) = 6.49; *p* < 0.001; 40.4 and 44.5, respectively, in women, *t*(3110) = 10.5, *p* < 0.001]. Among the individuals with high education, 13.8% scored moderate alexithymia (19.5% in men and 10.3% in females) and 4.2% scored clinical alexithymia (6.4% in men and 2.8% in women). Among the individuals with low education, 23.0% scored moderate alexithymia (27.1% in men and 19.2% in females) and 8.7% scored clinical alexithymia (9.8% in men and 7.6% in women). There was a very small negative correlation between alexithymia scores and age among the females (*r* = –0.09, *p* < 0.001) but there was no correlation among the males (*r* = –0.006, *p* = 0.77).

### Association between Dance Achievement and Alexithymia

**FIGURE 1:**
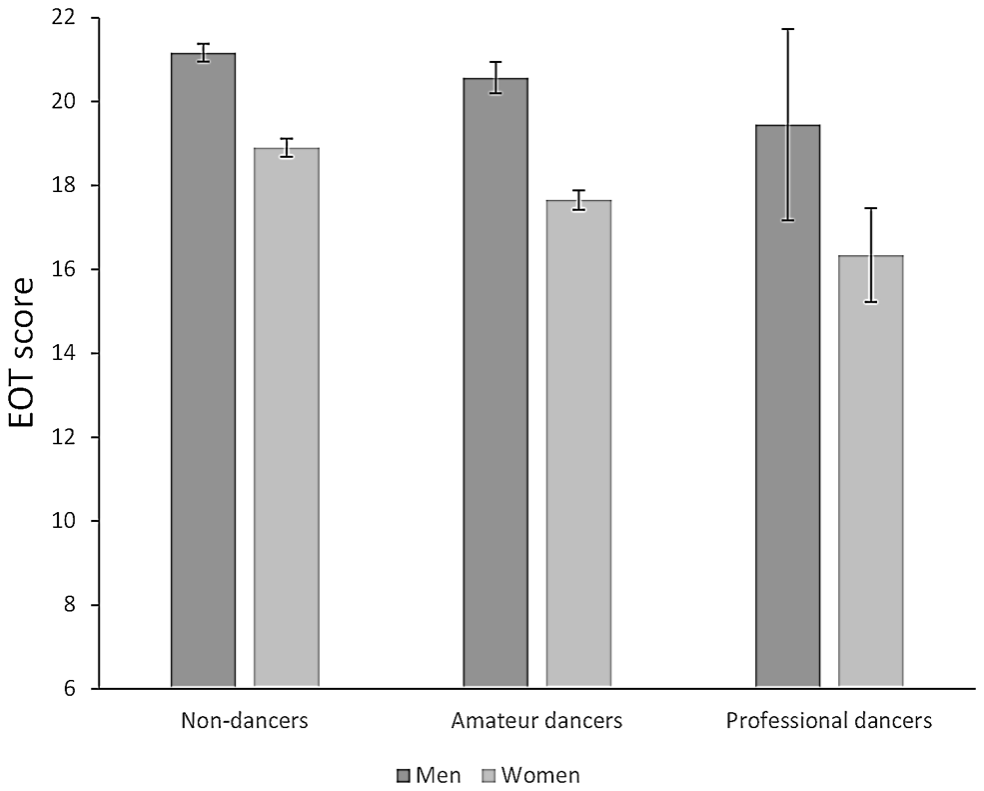
**Mean (95% CI) scores on the Externally Oriented Subscale in non-dancers, amateur dancers, and professional dancers**.

Among the female non-dancers 13.5% scored moderate alexithymia and 5.4% scored clinical alexithymia. The corresponding proportion were 12.5 and 3.2%, respectively, in amateur dancers and 10 and 2%, respectively, in professional dancers. Thus, there was a lower prevalence of alexithymia cases among the female dancers than among the female non-dancers. Among men, 22.7% of the non-dancers scored moderate alexithymia and 7.9% clinical alexithymia. The corresponding proportions were 22.6 and 7.5%, respectively, in amateur dancers and 15.0 and 0.0%, respectively, in professional dancers. Thus, while there was no difference in prevalence of alexithymia cases between male non-dancers and male amateur dancers, the male professional dancers had a much smaller prevalence (none scored clinical alexithymia). **Table 2** displays the mean scores of TAS-20 and its three subscales (measuring the different aspects of alexithymia) in non-dancers, amateur dancers, and professional dancers. In the analysis of the total scale there is a trend toward decreasing means in the three groups both in men and in women. With regard to DDF subscale, the trends were not consistent with our hypothesis. Concerning both the DIF scale and EOT scale the trends were in the expected directions, with decreasing mean with higher dance engagement. The findings are particularly strong for the EOT scale and this factor was in relation to dance in women differentiated consistently and significantly in both steps, from non-dancers to professionals as well as from non-dancers to amateurs. With the Bonferroni correction for significance, there was a significant difference in the expected direction among men (*p* = 0.006) with regard to EOT between non-dancers and amateurs. The difference in EOT score between male non-dancers and professionals was arithmetically larger than the non-dancer – amateur difference but failed to reach statistical significance. The small number of male professional dancers may have contributed to this (*n* = 20). Among women there were significant differences between non-dancers and professionals as well as between non-dancers and amateurs in the expected direction (both <0.001) with regard to EOT and also between non-dancers and amateurs with regard to DIF (*p* = 0.002). **Figure 1** shows the EOT scores in graphics.

**Table 2:** Mean (95% confidence interval) scores of the Toronto Alexithymia Scale (TAS-20) and scores on the sub scales measuring different aspects of alexithymia [Difficulty Identifying Feelings (DIF), Difficulty Describing Feelings (DDF), and Externally Oriented Thinking (EOT)] in non-dancers, amateur dancers, and professional dancers.

	Non-dancers	Amateur dancers	Professional dancers	*t*	*P*-value^a^	*t*	*P*-value^b^
*Men*	*N* = 1668	*N* = 577	*N* = 20				
TAS-20	46.6 (46.1–47.0)	46.3 (45.5–47.1)	42.1 (37.8–46.4)	–0.57	0.568	–2.00	0.046
DIF	12.6 (12.5–12.8)	12.5 (12.2–12.8)	11.0 (9.3–12.7)	–0.70	0.486	–1.87	0.062
DDF	12.7 (12.5–12.9)	13.2 (12.8–13.5)	11.6 (9.6–13.6)	2.02	0.044	–1.11	0.269
EOT	21.2 (21.0–21.4)	20.6 (20.2–20.9)	19.5 (17.6–21.4)	–2.77	0.006	–1.70	0.088
*Women*	*N* = 1678	*N* = 1385	*N* = 49				
TAS-20	42.5 (42.0–42.9)	41.0 (40.5–41.5)	39.7 (36.9–42.5)	–4.03	<0.001	–1.93	0.056
DIF	10.8 (10.6–11.0)	10.4 (10.2–10.6)	9.92 (8.89–11.0)	–3.15	0.002	–1.64	0.102
DDF	12.8 (12.6–13.1)	12.9 (12.6–13.1)	13.1 (11.8–14.5)	0.16	0.871	0.43	0.670
EOT	18.8 (18.6–19.0)	17.7 (17.5–18.0)	16.6 (15.4–17.8)	–6.77	<0.001	–3.50	<0.001

*Mean and 95% CI are adjusted for age and education level.*

*ANCOVA (adjusting for age and education levels) was used to compare the groups.*

*^a^Comparison between Non-dancers and Amateur dancers.*

*^b^Comparison between Non-dancers and Professional dancers.*

## Discussion

The results show that there is a significant association between the TAS subscale EOT and creative achievement in dance in female dancers compared to non-dancers. An increasingly favorable EOT score with increasing dance achievement corresponds to a more developed awareness of emotional processing and interpretation of emotions of others in dancers than in non-dancers. We observed no association in the expected direction, however, between the other factors DIF and DDF and dance achievement.

Dancers’ emotional regulation skills have been discussed as “embodied.” It has been suggested that this could imply that emotional skills can be trained in dancers in the same way as extensive training helps dancers achieve a high motor competence (Sevdalis and Keller, 2011). For example, studies have found decreased gray and white matter volume in brain areas associated with motor function in professional dancers relative to non-dancers (Haenggi et al., 2010). Expertise in a certain domain can be associated with a reduction of neural activity in the brain (associated with the given skill; Haslinger et al., 2004).

Our pattern of movement affects both our own perception of emotions and the way others perceive us (Bojner Horwitz, 2004). When externally oriented thinking decreases, less “penséé operatoire” is needed, according to Sifneos (1973). Sifneos related decreased “penséé operatoire” to increased skill in interpreting emotions of others. Therefore, we expected that dancers in this study would be more aware of their emotional processing than non-dancers. We did find that the female dancers in this sample were more aware of their emotional processing and showed a higher ability to interpret emotions of others than both male and female non-dancers. The findings on male dancers were in the same direction with a significant difference between non-dancers and amateurs.

In the literature, the term embodiment, and “embodied knowledge” is used to explain the body’s emotional feedback system where the motor system is linked to the cognitive affective system (Sheets-Johnstone, 1999; Koch and Fuchs, 2011). As discussed in another study (Landsman-Dijkstra et al., 2006), this expertise in dancing (skills) seems to increase access to embodied knowledge, which may facilitate identification of both adverse and empowering emotions.

The other subscales, difficulty to describe feelings DDF and difficulty identifying feelings DIF, were not significantly different in dancers compared to non-dancers. This means that dance achievement was not associated with ability to identify or verbalize feelings. External orienting of oneself emotionally may be a skill that dancers train through cognitively related mechanisms having to do with embodied knowledge, but it may also reflect mirror neuron networking and increased AOS (action observational system) activity in the brain (McGarry and Russo, 2011). In relation to Piaget’s model of different stages of cognitive development (Lane and Schwartz, 1987), it may be relevant to use dance movement as a tool for improving the symbolic representation of emotion for a more effective emotional regulation.

The majority of subjects who reported that they danced were amateur dancers. Only 3% were professional dancers. Professional dancing is defined as paid participation in a public dance performance. Dancers noted a higher frequency of exposure to or participation in cultural activities during childhood/adolescence and also had a higher general education level compared to non-dancers. This suggests that those involved in one type of cultural activity such as dancing are more likely to be involved in other creative areas. Early exposure to creative pursuits, of any type, may be important in establishing a long term interest in culture. Further studies could help to determine how these patterns are related to emotional regulation capacity.

As reported previously from our group (Theorell et al., 2014) and in line with other studies (Mattila et al., 2007), women had lower alexithymia levels than men. Individuals with higher education levels also had lower alexithymia scores. Learning abilities and learning skills may be related to strong emotional processes and related personal memories (Immordino-Yang and Singh, 2013) and accordingly important to evaluate in relation to other parts of the curriculum in the education system. Dance is often combined with music, and together these can induce emotions (Juslin and Sloboda, 2001) which may contribute to the decrease in the factor EOT in alexithymia.

Adams (2001), discusses emotional regulation strategies related to alexithymia affecting the ability to empathize. This may also affect capacity for empathy toward oneself. For example, a recent study showed that students that were asked to express themselves using body movements related to frustration association with writers’ block were better able to overcome these difficulties. The authors argue that this may be due to their increased capacity for self-directed empathy (Bojner Horwitz et al., 2013). Body movements and creative achievement through dance may contribute to increases in empathic behavior (Adams, 2001; Behrends et al., 2012).

In the results, we see a lower prevalence of alexithymia cases among the female dancers compared to the female non-dancers, and in the professional male dancers compared to the male non-dancers. Dance activity and training therefore seem to be involved in our emotional regulation system in which both performing and perceiving processes are linked to the mirror neuron activation system (Rizzolatti et al., 2001; Rizzolatti and Craighero, 2004). When creating a movement we activate neural areas which correspond to the limbic system (Umilta et al., 2001). This kind of emotional movement feedback system may help us to understand other persons’ feelings when they are in motion. This may also enhance our empathy for others.

The present analyses demonstrate a negative association between alexithymia and dance achievement in cross-sectional data. It is important to note that the causal mechanisms underlying this association may be complex. One possibility is that dance engagement has positive effects on emotional ability. However, the association could also be driven by a higher tendency to engage in dance in individuals with low alexithymia, or by unknown factors – e.g., genetic constitution – that simultaneously influence both alexithymia and dance achievement.

Dance has become a popular sport activity among young women and is well established as a training form among young men (O’Donovan and Kay, 2005), and may therefore play an important role as an “emotional regulator.” It may be useful to deepen our understanding of the association between other creative achievement skills and alexithymia in relation to social competence. One example of current research in this area is a controlled study of a combination of different cultural activities with burnout patients within the Swedish health care system, which showed that exposure to these activities was associated with a significant decrease in alexithymia scores (Bojner Horwitz et al., 2015).

### Strengths and Limitations

The study sample represents the Swedish population in the age group 27–54. In the sample there may be a slight overrepresentation of music-interested individuals as it was indicated during the recruitment that the overall research focus was on exploring musical engagement.

A structured interview for alexithymia (Caretti et al., 2011) has been tested in Italian samples, and acceptable correspondence was found between the self-administered questionnaire and the Toronto Structured Interview for Alexithymia (Grabe et al., 2014). De Gucht et al. (2004) examined the stability and validity of the three subscales of TAS-20 in clinical and non-clinical participants in France and showed that the three-factor structure of TAS-20 was confirmed across five samples. A study of almost 600 Iranian undergraduate students also confirmed the three-factor structure (Besharat, 2007). Säkkinen et al. (2007) also confirmed the three factor structure in a study of normal adolescents in Finland. Kooiman et al. (2002) have criticized the psychometric properties of TAS-20 but it seems that more problems arise in the use of TAS-20 in clinical samples (such as patients who somatize and psychiatric patients) than in non-clinical samples (as in the present study). Our conclusion from the literature is that TAS-20 and its three subscales can be used in normal populations and that there is acceptable correspondence with a standardized interview.

## Conclusion

The result from this study has shown a significant negative association between creative achievement in dance and the TAS subscale (EOT). Subjects with more dance experience had more favorable EOT scores. This corresponds to a more developed awareness of emotional processing and higher ability to interpret the emotions of others in dancers than in non-dancers. There were no consistent associations between the other factors DIF and DDF and dance achievement. Dance activity and training seem to be involved in the body’s emotional interplay with others. This suggests that there may be some clinical benefit of exposing those suffering from alexithymia to dance. However, we caution that we have only found evidence that these variables are correlated. It may well be the case that EOT scores are related to individual traits that also affect the propensity to be involved in dance. Nevertheless, our findings indicate that additional studies into the clinical benefit of dance for alexithymia are warranted.

## Conflict of Interest Statement

The authors declare that the research was conducted in the absence of any commercial or financial relationships that could be construed as a potential conflict of interest.
